# NaCl Dissociation Explored Through Predictive Power Path Sampling Analysis

**DOI:** 10.1021/acs.jctc.5c00054

**Published:** 2025-05-01

**Authors:** Konrad Wilke, Shuxia Tao, Sofia Calero, Anders Lervik, Titus S. van Erp

**Affiliations:** † Materials Simulation & Modelling, Department of Applied Physics and Science Education, Eindhoven University of Technology, 5600 MB, Eindhoven, The Netherlands; ‡ Department of Chemistry, Faculty of Natural Sciences and Technology, NTNU, Norwegian University of Science and Technology, 7941 Trondheim, Norway

## Abstract

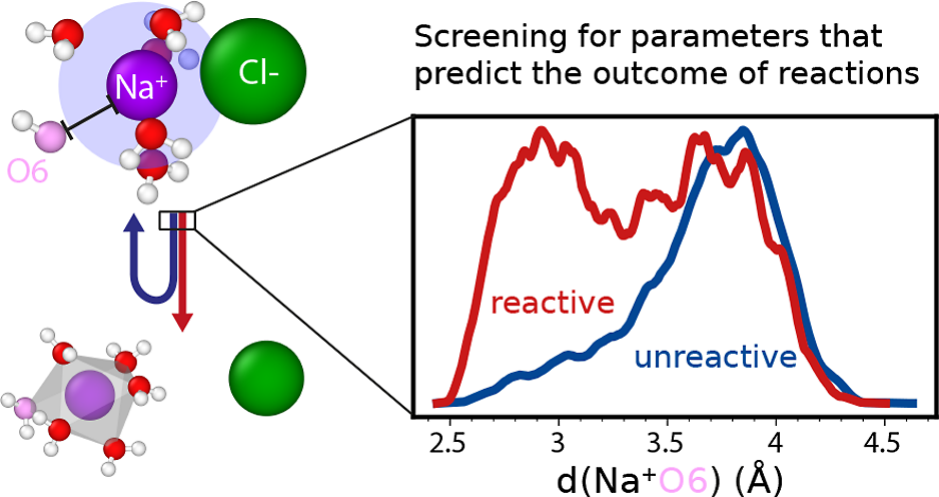

Utilizing the replica exchange transition interface sampling (RETIS) technique, we simulated the dynamics of sodium chloride dissociation in water. Subsequently, the resulting trajectories were analyzed using predictive power analysis (PPA), enabling the identification and quantification of collective variables (CVs) capable of forecasting the reaction occurrence. We improved the robustness of the PPA method by incorporating the Savitzky-Golay (SG) filter on integrated histograms, effectively avoiding the limitations associated with binning. Applying this adapted PPA method, the previously designed solvent parameters and distances from the index invariant distance matrix were assessed. This revealed that the sixth closest oxygen to sodium serves as an equally effective predictor as the best complex solvent parameter. The latter, however, required more knowledge and human intuition as an input for its design, while the former provided such intuition purely as an output. Through a comparable analysis, the chloride solvation shell appears to contain less predictive information. Employing a linear combination of several CVs can further enhance predictability, albeit at the expense of a reduced human interpretability.

## 1 Introduction

With the rapid advancement of computers, molecular dynamics (MD) simulations have facilitated the study of increasingly relevant processes on the atomic scale. Still, the accessible time and length scales of plain MD are generally not large enough for studying chemical and (bio)physical processes that typically evolve within an aqueous solution. These processes often involve water molecules actively participating in the reaction or require significant reorganization of the hydrogen bond network, adding to their complexity. This means that even if the relevant computational data can be generated, identifying and interpreting these types of relevant solvent motions remains challenging. Auxiliary simulation methods have, therefore, been developed for both sampling and analysis, which can be utilized in conjunction with MD.

Rare event simulation techniques address processes that occur infrequently per wall-time for standard MD.^1^ The time required to observe a single transition event in MD obviously varies depending on the system, the available computer hardware, and the level of theory, with ab initio MD being significantly slower than classical MD. Regardless of these parameters, simulation times for processes such as nucleation, chemical reactions, protein folding, and phase transitions can literally take centuries.

Sampling acceleration methods can be broadly categorized into two groups: those centered on free energy principles and those rooted in dynamics. The former, exemplified by umbrella sampling (US),^2^ metadynamics,^3^ and adaptive biasing force (ABF) methods,^4^ alters the energy landscape to facilitate exploration across reaction barriers, enabling derivation of thermodynamic properties. However, these approaches lack direct capture of the natural dynamics of rare events due to their artificial nature. In contrast, methods in the second category, such as transition path sampling (TPS),^5^ transition interface sampling (TIS),^6^ and replica exchange TIS (RETIS),^7^ generate trajectories that faithfully follow the true equations of motion and utilize Monte Carlo (MC) techniques to enhance barrier-crossings. These approaches not only yield insights into thermodynamics but also provide direct information about the actual dynamics of rare events.

In terms of analysis, the free energy methods naturally hinge on interpreting the free energy surfaces, which offer insights into (meta)stable states and barriers. However, the utility of this information is heavily contingent on the selection of collective variables (CVs). When the chosen CVs fail to capture dynamically relevant mechanisms, free energy barriers converge poorly, and even if they converge, the CV-dependent barriers are too low compared with the dynamically relevant reaction barriers. Consequently, the transition state theory (TST) often significantly overestimates reaction rates, while corrections like the reactive flux method^8^ face challenges due to very low transmission coefficients. Yet, if CVs are well chosen and capture the dynamics of the transition, the dimensionality of the CV space can, in principle, be reduced to a single optimal reaction coordinate (RC). In this scenario, the free energy projected onto this optimal RC yields the actual reaction barrier, with its maximum corresponding to the transition state dividing surface. Transitioning across this surface, toward either the product or reactant state, is assumed to proceed directly to those states without recrossing, rendering TST exact. The pursuit of this surface, such as through variational TST,^9^ is a central objective in these methods.

On the other hand, path sampling methods are often combined with committor analysis. Here, each phase point corresponds to a specific committor value, indicating the likelihood of the system entering the product state without passing through the reactant state. While committor probabilities are binary for deterministic dynamics, they range from 0 to 1 for stochastic dynamics. Points with the same committor value form isocommittor surfaces. While the true committor is defined in the phase space, it is common to compute the configuration space committor through velocity averaging, based on the assumption that the relevant dynamics behave effectively Brownian. The transition state dividing surface, if it exists, corresponds to the configuration-space isocommittor 1/2 surface. The latter, however, even exists when recrossings are unavoidable. Unfortunately, computing committor surfaces is computationally expensive. Approaches based on Bayesian statistics have been developed to accelerate this process,^10^ but they still require extensive additional simulations, even after a full rate evaluation via RETIS has been completed.

A related method utilized in this study is the predictive power analysis (PPA) method.^11,12^ PPA serves as a pure postsimulation analysis method, relying on the output of a RETIS simulation. The method assesses the distributions of the first crossing points with interfaces defined by specific values of the one-dimensional RC used in the RETIS approach. These crossing points are then characterized in terms of other CVs. Depending on the progression after the crossing, they are categorized as either “reactive” (*r*) or “unreactive” (*u*). By histogramming the two sets of points in the CV space, two distributions can be obtained, and the most predictive CVs are those that minimize the overlap between these distributions. This analysis can be performed for several interfaces and, in principle, can even be used to determine isocommittor surfaces without doing additional simulations.^11^ Note that the RC used here does not necessarily represent an optimal coordinate, and throughout this article we use the terms *reaction coordinate* and order parameter interchangeably.

In this article, we utilized RETIS simulations and subsequently applied PPA to probe the dissociation reaction of sodium chloride in water. While this transition is not exceptionally rare and could in principle be explored through brute force MD, albeit with reduced precision, it serves as a fundamental example where the solvent rearrangement profoundly influences the transition process.^13^ Consequently, understanding the mechanism of aqueous NaCl dissociation has been a focal point in numerous experimental^14^ and computational^15−18^ studies. Despite this, the dissociation mechanism remains not fully understood. Here, we leverage the NaCl model system not only to gain deeper insights into the mechanism but also to refine the PPA method for enhanced robustness, employing smoothed density approximations through Savitzky–Golay (SG) filtering to replace bin-based histograms. Additionally, we explore approaches for identifying CVs with significant predictive capacity that are intuitive and obtainable without a prior knowledge of the mechanism.

## 2 Methodology

### 2.1 Replica Exchange Transition Interface Sampling

Instead of generating a single lengthy dynamical trajectory from initial conditions as in plain MD, path sampling utilizes MD integration to advance both forward and backward in time from shooting points,^19^ resulting in numerous short trajectories. Within each temporal direction, trajectories are halted upon entering or exiting stable states, and each trajectory is subsequently accepted or rejected based on a Metropolis–Hastings MC scheme.^20,21^ Trial paths not fulfilling the ensemble’s criteria are always rejected. A randomly selected phase point of the last accepted trajectory is perturbed and served as a new shooting point. The MC detailed-balance scheme ensures that the same path distributions are generated as if they were extracted from an exceedingly long plain MD simulation. However, in path sampling, significantly more computational time is allocated to the barrier region, resulting in an exponential speedup relative to conventional MD.

The RETIS ensembles are based on both initial and final conditions, alongside a minimum progress requirement, evaluated via a series of nonintersecting interfaces. These interfaces correspond to hypersurfaces in the configuration or phase space, each linked to a fixed RC value λ_0_, λ_1_, ..., λ_*n*_. The initial and final interfaces, λ_0_ = λ_*A*_ and λ_*n*_ = λ_*B*_, delineate the boundaries of the reactant state *A* and product state *B*, respectively. This description marks one of the key advantages of the (RE)TIS method, as the RC can be arbitrarily simple if it can discriminate between state *A* and state *B* and is capturing the progress in between. The intermediate interfaces are strategically placed to optimize the computational efficiency. The path ensemble [*i*^+^] encompasses all trajectories starting by crossing λ_*A*_ toward the barrier region and concluding by re-entering *A* or entering *B*, while also mandating the crossing of λ_*i*_. The [0^–^] path ensemble explores the interior of state *A*. RETIS enhances TIS’s sampling efficiency by integrating replica exchange moves between these ensembles.^7^

Note that the use of replica exchange in RETIS differs fundamentally from conventional replica exchange techniques such as parallel tempering^22^ and Hamiltonian replica exchange.^23^ These methods rely on energy-based acceptance probabilities, which decay exponentially with the system size due to the extensive nature of energy, thereby requiring an increasingly large number of replicas for effective sampling. In contrast, RETIS performs exchanges between path ensembles defined solely by interface-crossing conditions without altering the temperature or the potential energy surface. As a result, RETIS achieves system-size-independent acceptance rates with the number of required interfaces determined only by the height of the reaction barrier.

After generating a significant number of paths in ensembles [0^–^], [0^+^], ..., [(*n* – 1)^+^], rates can be determined using the formula: . Here, *f*_*A*_ represents the frequency for the system to transition out of state *A*, and *P*_*A*_(λ′|λ″) denotes the conditional history-dependent probability for the system to reach λ′, given that it reached λ″ from state *A*. The flux *f*_*A*_ is calculated from the average path lengths in [0^–^] and [0^+^],^7^ while *P*_*A*_(λ_*i*+1_|λ_*i*_) is estimated from the fraction of sampled paths in the [*i*^+^] ensemble that cross the next interface λ_*i*+1_. The product of these probabilities yields the total crossing probability *P*_*A*_(λ_*B*_|λ_*A*_), representing the very slight chance that after exiting state *A*, the system manages to reach λ_*B*_ without revisiting state *A*. This estimation can be further enhanced through path reweighting techniques,^11,24^ incorporating *P*_*A*_(λ_*i*+*j*_|λ_*i*_) with *j* > 1 into the estimation for the overall crossing probability based on the weighted histogram analysis method (WHAM).^25^

### 2.2 Predictive Power Analysis

RETIS ensembles include trajectories that exhibit different levels of progress along the RC, which can be studied to understand why some trajectories advance further, potentially reaching state *B*, while others do not. One approach is to visually inspect molecular simulation movies to identify the molecular patterns and behaviors. However, a more systematic data-driven analysis can be performed using the PPA method.^11^ The method provides a quantitative measure for assessing how well a CV or set of CVs, distinct from the RC, can predict whether trajectories that reach a specified crossing interface λ^c^ will also reach a defined reaction interface λ^r^. These interfaces can, but do not have to, correspond to any of the RETIS interfaces and can be positioned: λ_*A*_ ≤ λ^c^ ≤ λ^r^ ≤ λ_*B*_.

Let Ψ = (CV_1_, CV_2_, ..., CV_*m*_) represent the vector in the CV space, when *m* CVs are selected as additional descriptors for the reaction. From all the trajectories generated by the RETIS simulation, the trajectories that cross λ^c^ are considered and weighted^11^ such that the distribution of paths corresponds to the statistical distribution that would result from cutting out the segments of an infinitely long MD simulation. Subsequently, Ψ values for the points of first crossing with λ^c^ are considered for those paths, resulting in a distribution *t*(Ψ) at λ^c^. This distribution is further split into two distributions: the points lying on paths that also cross λ^r^ and those on paths that do not. These two distributions are denoted *r*(Ψ) and *u*(Ψ) with *r*(Ψ) + *u*(Ψ) = *t*(Ψ).

Based on these distributions, the predictive capacity  provides a measure of how effectively the information about Ψ predicts whether the transition to λ^r^ occurs after crossing λ^c^. On average, the probability of this transition equals . However, if the chosen CVs are well correlated with the success of the transition specific regions in the CV space should have a significantly higher probability, i.e., . Nevertheless, regions at the λ^c^ interface with a high transition probability should not be overly emphasized if only a small fraction of the actual transitions pass through them. Therefore, the predictive capacity is based on a weighted average of *r*(Ψ)/*t*(Ψ), weighted by the fraction of reactive trajectories that pass through Ψ: . As shown in ref (11), this leads to the following expression
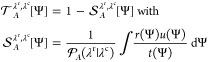
1where  is the overlap integral. One can show that , where the lower and upper limits are obtained for least and most predictive CVs, respectively.

### 2.3 Savitzky–Golay Fitting of Integrated Histograms

While eq 1 provides a conceptual measure of predictive power that is broadly applicable to any hypothesis set of CVs, its practical implementation can be tricky and, if done incorrectly, can lead to erroneous conclusions. Typically, estimating distributions requires some form of histogramming, where data is partitioned into intervals along each CV known as bins. Each bin corresponds to a hypercube in the CV space with a specific range, and the number of data points falling within each bin is tallied. The selection of the bin size is critical, as it influences the appearance of the histogram and the fidelity of the distribution representation. Bins that are too wide may obscure important details, while overly narrow bins can produce noisy histograms. This presents challenges for the PPA approach, as the binning method transforms the overlap integral into the following summation
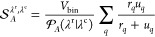
2with *q* being the bin-index and *V*_bin_ the bin-volume. With too few large bins, the resolution may fail to capture the distinctiveness between the shapes of the *r* and *u* distributions, resulting in an underestimation of the predictive capacity. Conversely, if the bins are too small, each bin may contain either zero or one data point of type *r* or *u*, leading to a zero overlap and falsely suggesting that . In this article, we introduce an approach to alleviate this problem for the *m* = 1 case, i.e., for a one-dimensional Ψ, based on fitting integrated distributions using the SG filter.

Let *R* and *U* denote the integrated distributions for *r* and *u*, respectively, defined as *R*(Ψ) = ∫_–∞_^Ψ^*r*(Ψ^′^) dΨ^′^ and similarly for *U*. Consider the CV values of the simulated data points {CV(1), CV(2), ...}, ordered in increasing order, each point either belonging to the *r* or *u* category. Due to the WHAM procedure, these data points are not equally weighted. For each data point *i*, part of *r*, we can estimate *R*(CV(*i*)) as *R*(CV(*i*)) = ∑_*j*∈*r*_^*i*^*w*_*j*_/*W*, where *w*_*j*_ is the weight of data point *j* and *W* is the sum of all (both *r* and *u*) weights. The integrated distributions can hence be determined for all values on the CV axis represented in the data set. Via a simple linear interpolation between these points, the *R* and *U* curves are initially fitted on a regular grid using an abundant number of grid points between the CV extrema. Subsequently, we applied an SG fit using a second-order polynomial with a window length *l*_w_ covering approximately 1/16 of the CV range, rounded to the nearest odd integer, as required by the method. To eliminate boundary effects in the SG fitting, the integrated curves are extended on both sides with horizontal plateaus: on the left, at height zero and, on the right, at the final value, each spanning one-quarter of the range of the CV. From this, the derivatives—and consequently the *r* and *u* curves—were derived, sidestepping the binning approach.

**Figure 1:**
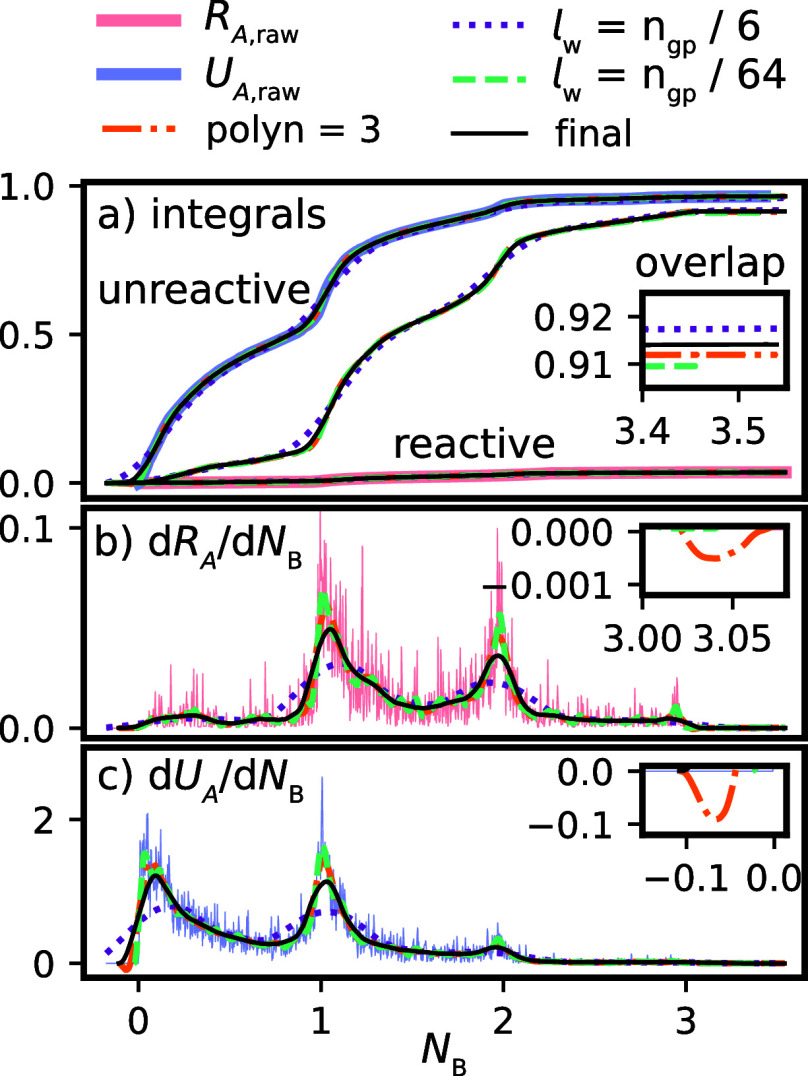
Analysis of SG fitting procedure. (a) Raw integrated distributions for *N*_B_ (the number of bridging waters^15^), the fitted curves using different SG-filter settings, and the progression of the resulting overlap integral  (eq 1) as a function of the upper integration boundary. The number of grid points, *n*_gp_, to compute the raw distributions was set to 10,000. All SG-fitted curves overlap with the raw curves *U* and *R* within the thickness of the lines, except when the window length exceeds one-sixth of the CV range. However, when taking the derivatives, details that are invisible in the top panel become apparent in the lower panels. The inset zooms in on the final value of the overlap integral, demonstrating that all SG fits yield an  value of approximately 0.91, highlighting the robustness of the approach. (b,c) *u* and *r* distributions obtained by taking the derivatives of the fits. Notably, even the raw fits based on piecewise linearization without an SG filter, though very spiky, do not show any zeros in the relevant range—unlike what would occur with direct histogramming based on 10,000 bins. The SG fit with a polynomial order (polyn) of 3 is not entirely physical, as the estimated distributions can become negative (see the insets for a detailed view). The final SG setting was based on a polynomial of second order with a window length equal to *l*_w_ = *n*_gp_/16, which provides sufficient details without producing overly spiky overfitted distributions.

The relative window length of 1/16 was based on preliminary analysis, as it provides sufficient detail while avoiding overfitting the noise (Figure 1). Still, the overlap does not drop to zero despite more spiky distributions when the window size is taken too small. Using a polynomial order within the SG fit larger than 2 could lead to distributions with negative amplitudes, which was, therefore, disregarded.

### 2.4 Collective Variables

The PPA method, in principle, solely provides a measure of how valuable a CV or set of CVs is for predicting the transition to λ^r^ when λ^c^ is crossed for the first time since exiting state *A*. It does not provide a direct strategy to identify the relevant CVs themselves. In a previous study,^12^ a small set of potentially relevant CVs was explored to study the mechanism of water autoionization using decision trees based on a large input set of CVs. The most prominent CVs were then combined into an Ψ vector containing up to four CVs and tested for their predictive capacity. The set of CVs with the highest predictive capacity can be further analyzed, for example, by assessing whether the dimensionality of the Ψ vector can be reduced without significantly decreasing the predictive capacity. It can be shown that this ultimately provides an approach to obtain the committor based on the RETIS output alone, without the need for additional molecular simulations targeted to get the committor.^11^

However, the binning problem, where bins have too few data points, becomes even more severe in a high-dimensional space, as the number of bins scales exponentially with the dimension of the Ψ vector. Additionally, our approach to mitigate this issue (see Section 2.3) is practical only for a one-dimensional CV vector. Therefore, we opted for a different machine learning (ML) strategy in which linear combinations of a few CVs directly provide a one-dimensional Ψ vector, whose predictive capacity can be measured using the SG approach. Both the combination of CVs and their coefficients are optimized with respect to the predictive capacity for a particular set of λ^c^ and λ^r^ using a simulated annealing (SA) approach.^26^ The number of CVs in the linear combination was restricted for increased interpretability and because we observed that the predictive capacity only marginally improved by relaxing this constraint.

As inputs, we included a set of CVs that were previously designed and analyzed for their ability to describe the configuration-space isocommittor 1/2 surface,^15^ and additionally incorporated the index-invariant distance matrices (IIDMs).^27^ The latter requires only the assignment of an anchor atom. From a full distance matrix, where the entry *M*_*ij*_ at row *i* and column *j* indicates the distance between atom *i* and *j*, the matrix is first sorted by rows, initially by species and then by distance to the center ion. Subsequently, columns within each row are sorted in the same manner, first by species and then by the distance to the corresponding row atom. This allows for truncating the matrix to a desired dimension, effectively capturing the local environment’s signature and focusing on the nearby orientation around the anchor atom and its nearest neighbors without necessitating a high-dimensional description. To indicate the IIDM distances, we use the notation XY*i*Z*j* to represent the distance between atoms of species Y and Z, where X is either Na^+^ or Cl^–^, indicating the anchor ion. In this notation, Y*i* refers to the *i*th closest atom of type Y to X and Z*j* represents the *j*th closest neighbor atom of type Z to Y*i*. To denote the distance between an anchor atom and its *i*th closest Y atom, we simply use XY*i*.

## 3 Computational Details

The transition paths between the associated and dissociated ion pair were sampled using the RETIS method, implemented in PyRETIS 2.5,^28,29^ interfaced with GROMACS version 2021.1^30^ for performing the MD steps. The system consisted of 908 TIP4P water molecules^31^ and one ion pair of NaCl in a cubic box with periodic boundary conditions. The integration of motion was carried out with the OPLS-AA force field^31^ using the velocity Verlet algorithm^32^ with a time step of 2 fs. Electrostatic interactions were computed through the smooth particle mesh Ewald (PME) of the fourth order with a grid spacing of 0.16 nm.^33^ A Verlet cutoff scheme and 3D-Ewald geometry are applied, with the electrostatic and van der Waals cutoff radii set to 1.4 nm.

To equilibrate the system and determine the box dimensions, a plain MD simulation was run in the *NPT* ensemble using a velocity-scale thermostat^34^ set at 300 K with time constant for a coupling τ_*t*_ of 0.1 ps and a Parrinello–Rahman barostat^35^ at 1 bar with a τ_*p*_ of 0.2 ps and an isotropic pressure coupling. After an initial stabilization phase, the size of the cubic simulation box oscillated around an average of 30 Å, which was then fixed for the RETIS simulation. The RETIS simulations were run by using deterministic *NVE* dynamics with uniform *NVT* shooting. In this approach, any time slice of the latest accepted path has an equal probability of being selected as a shooting point, after which the velocities are completely regenerated from a Maxwell–Boltzmann distribution corresponding to the target temperature. By doing so, the total energy within a trajectory is conserved but the energies differ between the sampled paths. This situation mimics a molecular system that is weakly coupled to a thermostat such that changes in the molecular system’s energy are observable on a time scale that is substantially larger than the average path length. The advantage over fully thermostated dynamics is that the dynamics become more physical because thermostats perturb the true Newtonian equations of motion and also remove some element of randomness, making the PPA method potentially more insightful. Moreover, another argument for deterministic dynamics is that the PPA method measures a time correlation between the crossing of λ^c^ and the subsequent crossing of either λ^r^ or λ_*A*_. If λ^c^ is close to λ^r^ or λ_*A*_, then the time interval between these events can be shorter than the velocity autocorrelation time, meaning that any artificial modification of the momenta could have an impact. However, our preliminary simulations, which were conducted with thermostated dynamics, showed no noticeable differences in predictive capacity compared to our production runs using NVE dynamics.

The RETIS runs were configured with a swapping and shooting frequency of 0.5. During the shooting moves, the PyRETIS program interacts with the GROMACS engine at regular intervals of 5 MD time steps to update the RC λ and instruct the engine to store the configuration. Consequently, all analyzed trajectories had an effective time step of 10 fs. For the RC, we chose the interionic distance between Na^+^ and Cl^–^ as it is a simple measure that continuously increases between the associated state *A* and the dissociated state *B*. Based on some preliminary runs, the RETIS interfaces along λ were set at the positions: [3.2, 3.4, 3.6, 3.8, 4.1, 7.0] Å, where we aimed for local crossing probabilities, , to lie between 0.2 and 0.5, which is considered to be close to optimal.^36^

The rate constant *k*_*AB*_ was calculated based on the full set of trajectories. For the PPA method, a subset comprising every 10th path in the MC chain was extracted for data efficiency. The full run included 48,378 unique, accepted paths in the [i^+^] ensembles, and 25,453 in the [0^–^], encompassing 3207 unique full transitions from λ_*A*_ to λ_*B*_. The subset used for PPA contained 20,977 unique paths, including 2538 transitions. Average path lengths for the ensembles ranged from 822 to 10,720 fs. The total number of MD steps, including rejections, was 1.8 × 10^8^. This resulted in a total wall time of approximately 8 days using 24 threads on Intel Xeon E5–2687W CPUs, distributed equally across 6 ensembles.

**Figure 2:**
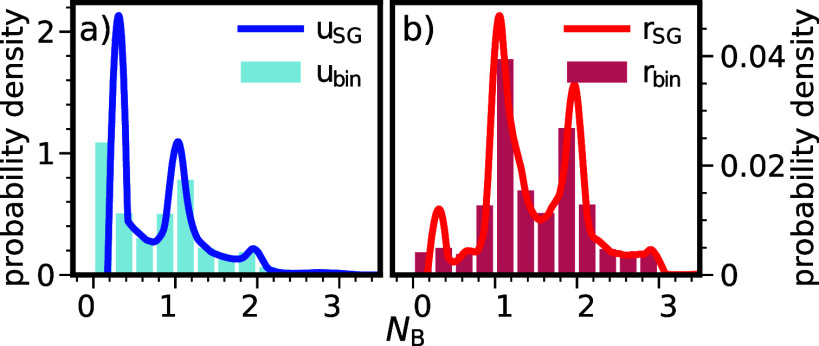
Comparison of the *u*(*N*_B_) and *r*(*N*_B_) distributions obtained using the SG filter approach versus standard binning.

Using the approach in Section 2.3, the integrated distributions were mapped to a regular grid with 2000 grid points between the maximum and minimum sampled values of the considered CV. We extended the integrated distributions by 500 grid points in both the maximum and minimum direction. They were then smoothed using a second-order polynomial SG filter with a window length of 125, the closest odd number to 2000/16. Figure 2 compares the *r* and *u* distributions obtained using standard binning and the SG approach for λ^c^ = 3.2 Å and λ^r^ = 7.0 Å, with the CV defined as *N*_B_, the number of bridging water molecules.^15^ The SG approach demonstrates a substantially improved resolution, ultimately enabling a more accurate, robust, and reproducible calculation of the predictive capacity, as described by eq 1.

In our effort to find the best, yet interpretable, CV, we analyzed a set of 21 CVs from Mullen et al.^15^ This set includes the number of bridging water molecules (*N*_B_), eight different angles between the solvent and NaCl, and ten water densities around the ion axis. Additionally, we evaluated two generalizations of the IIDM,^27^ using either the sodium (Na^+^) or chloride (Cl^–^) ion as the anchor atom. For both ion centers, we calculated matrices containing the respective counterion, the 15 closest oxygen atoms, and the 30 closest hydrogen atoms, resulting in a total of 4636 unique distances. These variables, along with their linear combinations of up to three terms, Ψ = αCV_*i*_ + βCV_*j*_ + γCV_*k*_, constrained by |α| + |β| + |γ| = 1, were tested as one-dimensional CVs. Note that this constraint does not limit the possible range of CVs, as an overall scaling of Ψ has no effect on its predictive capacity. Each CV was made dimensionless, with distance-based CVs divided by 1 Å and density-based CVs divided by nm^–3^. In the process of linear combinations, the input CVs were picked based on their individual predictive power and combined with the combinations function of the itertools Python library. The coefficients were randomly altered, constrained by α,β,γ ∈ [−1,1], using MC moves within the dual_annealing scheme^37^ from the SciPy Python library.^38^ Finding the optimal combination was a 2-step process of a screening step and a local search step. The screening was performed with an initial temperature of 26,150, with the maximum number of optimization iterations set to 200 when using 2 CVs in the linear combination, and 1000 iterations when 3 CVs are included. The most promising candidates identified during the screening phase were further refined through a local search to minimize the overlap.

## 4 Results and Discussion

### 4.1 Rates and Crossing Probabilities

**Figure 3:**
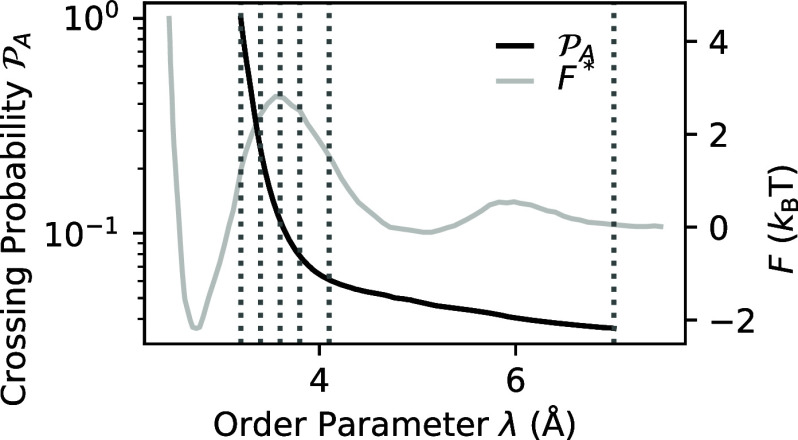
Crossing probability  against the order parameter compared to the Helmholtz free energy *F* calculated by Ballard and Dellago.^16^

Figure 3 illustrates the crossing probability, , as a function of λ, ranging between λ_*A*_ = 3.2 and λ_*B*_ = 7 Å, obtained from the RETIS simulation. The absence of a horizontal plateau at the end suggests a diffusive ion drift and a moderately low barrier for the reverse association transition. Consequently, the rate constant *k*_*AB*_ exhibits a weak dependency on the somewhat arbitrary definition of the product state boundary λ_*B*_, indicating that this process lies on the borderline of the rare event category. This stands in contrast to other processes, such as water dissociation^12^ or silicate condensation reactions,^39^ which unequivocally fall within this category and display crossing probabilities ending with a completely flat plateau. Based on the defined boundaries, we obtained a total crossing probability of  with a flux of *f*_*A*_ = 1728 ± 3 ns^–1^, resulting in a dissociation rate of *k*_*AB*_ = 6.5 ± 0.4 ns^–1^.

### 4.2 Single Input CVs

**Figure 4:**
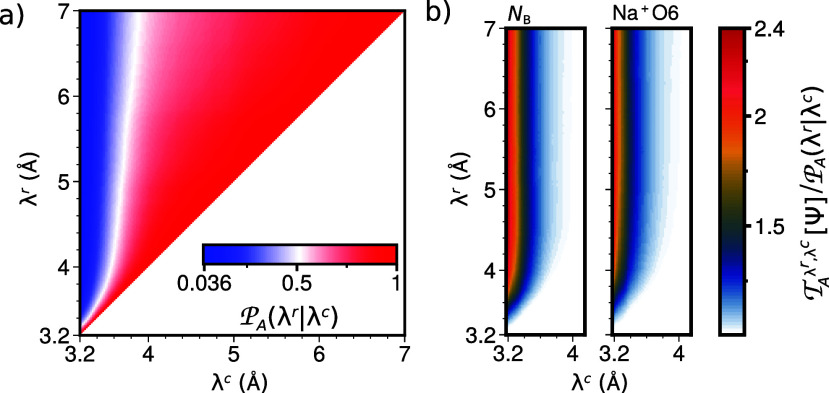
(a) Crossing probability  and (b)  of the predictive section for bridging water *N*_B_ and the sixth closest oxygen Na^+^O6 on a grid of *n*_grid_ = 200 from 3.2 to 7.0 Å along both λ^c^ and λ^r^.

Figure 4a presents a 2D visualization of the crossing probability  for all possible (λ^r^, λ^c^) pairs, with a grid spacing of dλ = 1.9 × 10^–2^ Å. It is worth noting that this grid spacing in λ^c^ and λ^r^ can theoretically be arbitrarily small, as the computations of  and  are unaffected by the aforementioned binning problem. The 2D plot in Figure 4a directly follows from the results of Figure 3 using . From this figure, it is evident that the crossing probability approaches unity when λ^r^ is close to λ^c^ or when λ^c^ surpasses the dissociation barrier. In this region, the predictive capability is less valuable, as  is bounded between  and 1 for any set of CVs.

Outside this region, the predictive capacity can be significantly higher than the crossing probability as is clear from Figure 4b,c where we showed the predictive capacity relative to the crossing probability,  for the best single input CV from ref (15) and the best distance from the IIDM analysis.

The number of bridging waters *N*_B_ and the distance between the sodium ions and its sixth closest oxygen, Na^+^O6, show predictive capacities that are up to a factor of 2.4 larger than the crossing probability. Here, *N*_B_ roughly describes the number of water molecules simultaneously part of the first solvation shells of both ions.^15^ It can be a fractional number, as it is based on summing the values of an indicator function, where the input argument is the distance between water molecules and ions, taken over all water molecules. The indicator function smoothly transitions from one at short distances to half at the solvation radius and to zero at large distances. Both the solvation radius and the steepness of this transition require adjustment, indicating a significantly higher level of human-driven design compared to Na^+^O6, which is simply a distance. However, due to the shuffling of the matrix in the IIDM approach, Na^+^O6 also contains some collective information, not just the distance between two atoms. This is because it represents the distance from Na^+^ to the sixth closest oxygen, thereby indicating that five oxygens are even closer, while all others are further away. Hence, a low value of Na^+^O6 can be interpreted as describing a compression of a solvation shell of six oxygens.

**Figure 5:**
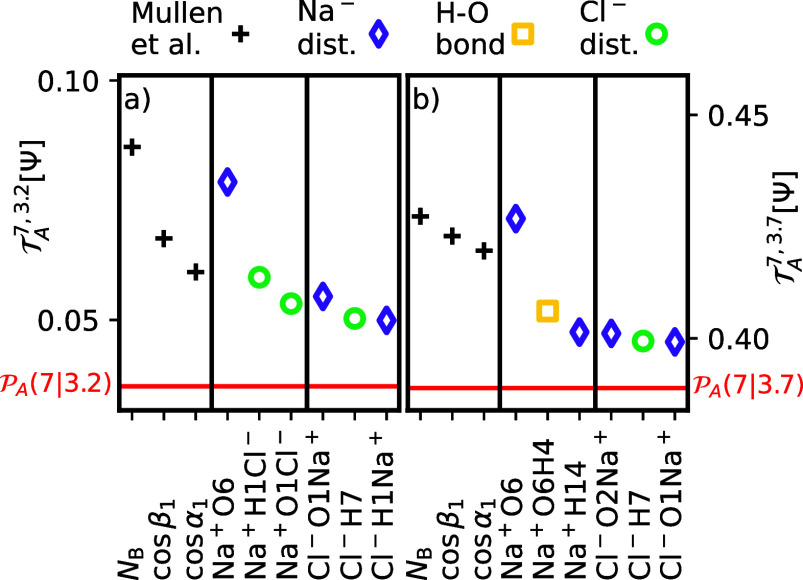
Input CVs from the complex solvent parameters of ref (15) and the ion-centered IIDMs with the highest predictive capacity  with (a) λ^c^ = λ_*A*_ and λ^r^ = λ_*B*_ and (b) λ^c^ = λ_*A*_ and λ^r^ = λ_TS_. The solvent parameters describe the number of bridging waters *N*_B_, the largest cosines of α = ∠Na–Cl–H and β = ∠Cl–Na–O.

Figure 5a provides an overview of the input CVs with the highest predictive capacity for the case λ^c^ = λ_*A*_ = 3.2 Å and λ^r^ = λ_*B*_ = 7 Å.

The number of bridging waters, *N*_B_, exhibits the highest predictive power, aligning qualitatively with the findings of ref (15), which indicates that this parameter provides the best description for the committor alongside λ. However, Na^+^O6 performs only slightly worse. Additionally, the angular parameters cos β_1_ and cos α_1_ from the Mullen set^15^ perform reasonably well, surpassing the interionic density ρ_*m*3_. From the IIDM, the best parameters in Figure 5a are either species that can be located in one of the first two solvation shells (Na^+^O6, Cl^–^H7) or that contain information about the interionic structure. This indicates that those are the areas that contain the significant mechanistic information. Na^+^H1Cl^–^ serves as the second-best input CV, although it is substantially less effective than Na^+^O6, yet still better than any distance from the Cl^–^-centered matrix. This suggests that the solvent structure around the positive sodium ion is more crucial for initiation of the dissociation than the solvent structure around the negative chloride ion.

This conclusion is further supported by the fact that the best Cl^–^-centered IIDM, Cl^–^O1Na^+^, also involves the sodium ion. It is also noteworthy that the IIDM with Na^+^ as an anchor has nearly the same predictive capacity for the full λ_*A*_ → λ_*B*_-transition as the best complex solvent parameter, *N*_B_. Shifting λ^c^ to λ_TS_ = 3.7 Å, smaller predictive capacities relative to the crossing probability are anticipated, which are consistent with the findings in Figure 4b. All three of the best Na^+^-anchored parameters refer to species that are important for migration processes among the respective first and second solvation shells. Remarkably, the second best parameter Na^+^O6H4 is a measure for the compression of hydrogen bonding around the Na^+^O6 oxygen. The Cl^–^ IIDM is still containing similar types of distances as the front runners, compared to the results with λ^c^ = λ_A_ as a starting point. Ge et al. extensively studied Cl^–^ in aqueous solution, demonstrating that its first solvation shell, similar to Na^+^, consists of 5–6 water molecules.^40^ However, different size ratios and water orientations result in a variety of configurations and a high entropy around Cl^–^. Our simulations reflect this behavior, showing a reduced predictive power for elements of the Cl^–^-centered IIDM.^40^

**Figure 6:**
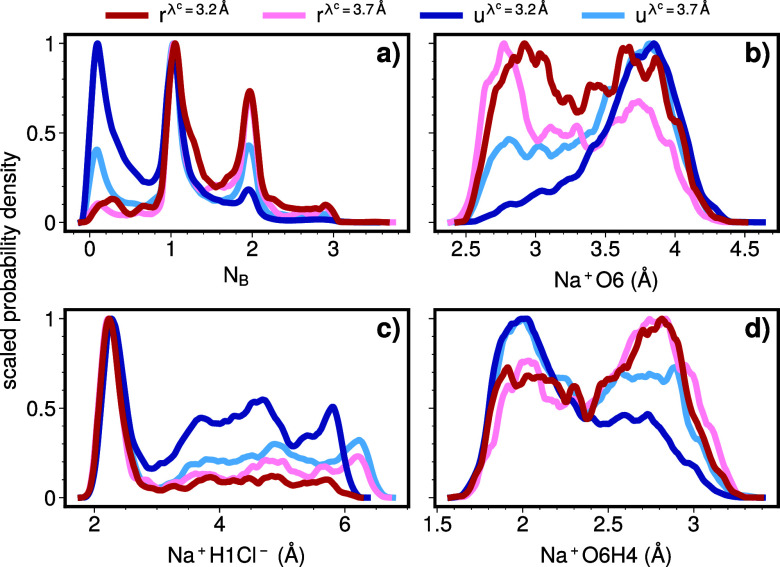
(a−d) *u*- and *r*-distributions of mechanistically relevant CVs at the start (3.2 Å) and at the reaction barrier (3.7 Å) with λ^r^ = 7 Å. The distributions are scaled so that their maximum value is set to one.

Figure 6 shows the corresponding *r*- and *u*-distributions that lead to the predictive capacities of Figure 5, i.e., for (λ^c^, λ^r^) = (λ_*A*_, λ_*B*_) and also for (λ^c^, λ^r^) = (λ_TS_, λ_*B*_) with λ_TS_ being the transition state or local maximum of the free energy barrier (see Figure 3). This approach allows us to compare reactive trajectories with the most common unreactive ones and to gain insights into the factors that differentiate fully reactive trajectories from nearly reactive ones—those that reach the transition state but do not proceed to the product state.

Many CVs with a high predictive capacity describe the region around the ion axis. For instance, in Figure 6a, we observe that reactive trajectories tend to have high values for *N*_B_ compared with unreactive trajectories, highlighting the importance of intervening water molecules in stabilizing the charge separation between the ions. If we switch the crossing interface to λ^c^ = λ_TS_ = 3.7 Å, both the *r*- and *u*-distributions will change due to molecular movement and the evaluation occurring later along the trajectories. Additionally, the *u*-distribution will also change because trajectories that do not reach λ_TS_ are eliminated from the analysis. By comparing the λ^c^ = λ_*A*_ and λ^c^ = λ_TS_ cases, we can conclude that the first effect has little influence on the change in the *N*_B_ distribution, as evidenced by the fact that the relative peaks in the *r*-distribution at *N*_B_ = 1 and *N*_B_ = 2 do not significantly change. The second effect, however, is clearly visible, as the *u*-distribution becomes nearly identical to the *r*-distribution. This also indicates that a high *N*_B_ value is strongly indicative of reaching the transition state but not necessarily of reaching the product state once the transition state has been achieved.

We can draw an aligning conclusion from Figure 6c, where the probability of a reaction is low, if the closest H to Na^+^ is not close to Cl^–^ at λ^c^ = λ_*A*_. In the nearly reactive transition state, the reactive and unreactive distributions are almost indistinguishable. This behavior contrasts sharply with that of Na^+^O6, as shown in Figure 6b. At λ^c^ = λ_*A*_, the *r*-distribution exhibits two peaks of equal height, whereas the peak at short distances is nearly absent in the *u*-distribution. Upon reaching λ^c^ = λ_TS_, both distributions develop double peaks, with the peak at low distances shifting further downward and becoming dominant in both distributions. The change in the *r*-distribution arises solely from molecular motion along the same reaction paths, while for the *u*-distribution, some paths from the λ^c^ = λ_*A*_ case are no longer included in the distribution. The shift in the *r*-distribution suggests that to transition from λ_*A*_ to λ_*B*_, one must either begin with a contracted solvation shell or possess the ability to quickly contract the solvation shell. This capability could potentially be quantified by using an additional CV, suggesting that combining information from multiple CVs may enhance predictive power.

Furthermore, Figure 6d reveals information regarding the local surroundings of the Na^+^O6 oxygen. Here, H4 is the second closest extra-molecular hydrogen. In both cases, λ^c^ = λ_*A*_ and λ^c^ = λ_TS_, the unreactive distribution peaks at shorter distances, in contrast to the reactive case, which is more likely when the Na^+^O6H4 distance is large. Interestingly, we can identify the oxygen with which the Na^+^O6H4 hydrogen forms a bond. This bond is typically formed with either the second or third closest oxygen to the Na^+^O6 oxygen. However, neither Na^+^O6O2 nor Na^+^O6O3 exhibits significant predictive power. This suggests that Na^+^O6H4 is indicative not of the position but rather of the orientation of water molecules around the Na^+^O6 oxygen.

To gain deeper insights into the evolution of the Na^+^O6 oxygen, we extended our analysis beyond the *r*-distribution, which considers only the distance to Na^+^, by examining the corresponding 2D spatial distributions around the Na^+^–Cl^–^ axis. By overlaying this spatial distribution on top of the multioxygen distribution of the 15 closest oxygens, we observe how the sixth closest oxygen to Na^+^ relates to the other oxygens surrounding the ion pair. Furthermore, we tracked the progression of these 2D distributions throughout the reaction as the ionic separation distance increases.

**Figure 7:**
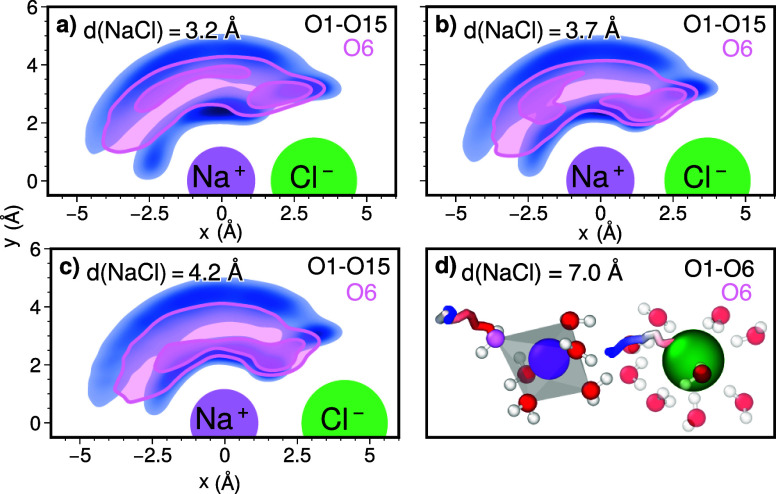
Spatial probability density distributions of Na^+^’s 15 closest oxygens, shown around the Na^+^–Cl^–^ axis. The probability density to find an O in the dark areas is 10–45%, 1–10% in the light areas, and <1% in the white space. (a) Start of the reaction, where the ionic distance is λ_*A*_ = 3.2 Å; (b) transition state at λ_TS_ = 3.7 Å; and (c) state beyond the transition state, where the ionic distance has increased to 4.2 Å. The multioxygen distributions are shown in blue, while the distribution of the Na^+^O6 oxygen is highlighted in red. (d) Exemplar dissociated state, showcasing a pseudo-octahedral alignment of the six closest oxygens and the coordinated movement of Cl^–^ and the Na^+^O6 oxygen over time.

The underlying multioxygen distribution reveals the presence of two distinct water shells. Initially, the Na^+^O6 oxygen predominantly resides in the outer shell (see Figure 7a). As the reaction progresses, Figure 7b,c reveals a shift of the Na^+^O6 oxygen toward the inner shell, occurring in specific regions around Na^+^, and by the end of the reaction, it is predominantly located in the inner shell. In the final stage, the distances between the sodium ion and its six closest oxygen atoms converge, forming a pseudo-octahedral arrangement around Na^+^ (see Figure 7d). Additionally, the Na^+^O6 oxygen predominantly enters the inner shell from the side opposite to the departing Cl^–^ ion, suggesting a coordinated push–pull interaction between the two.

These findings align with previous computational studies Belch et al. and recent experimental results of Persson.^14,41^ Jung et al. studied the reverse process of the assembly of ion pairs in water and outlined the importance of the inner-shell water rearrangement. They report the necessity of water molecules opening up the space around the cation for the anion.^18^ This finding is complementary to our results, as we can take from the visualization that the sixth closest water will close the space and that the anion opens by migrating away.

### 4.3 Linear Combination of Input CVs

**Figure 8:**
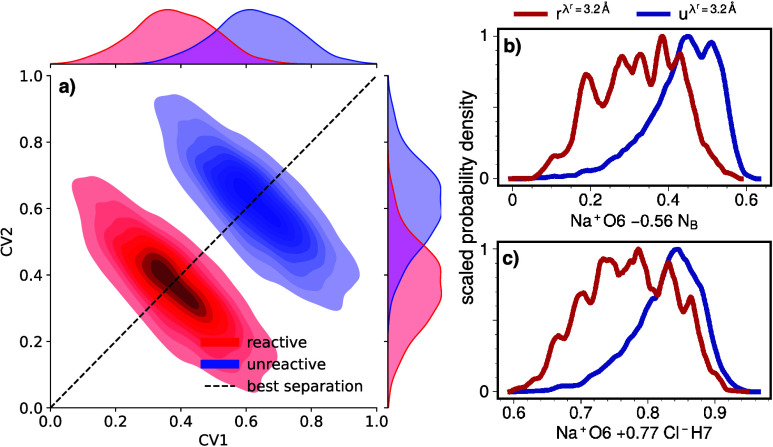
Illustration of the enhanced predictive power achieved by using linear combinations of CVs. (a) Two-model CVs and how their linear combination can reduce the overlap between the *r*- and *u*-distributions. (b,c) *u*- and *r*-distributions, respectively, for the optimal combination of two general CVs and for the best combination based solely on IIDM elements, at λ^c^ = 3.2  Å and λ^r^ = 7  Å.

Figure 8a illustrates the potential of using linear combinations of CVs as inputs to uncover mechanistic links and gain deeper insights into the reaction mechanism. To achieve this, we employ a machine learning approach that examines all possible linear combinations, up to three, as discussed in Section 3, with  serving as the loss function to be minimized using simulated annealing.

Figure 8b,c showcases, respectively, the best overall and the best linear combination derived solely from the IIDM. In both cases, features of the individual CVs remain discernible, but the resulting *r* and *u* distributions exhibit less similarity to each other than is seen in Figure 6. Comparing the optimal combinations in Table 1 provides a more comprehensive understanding of the mechanism, as it highlights the key information relevant at each stage of the reaction.

At λ^c^ = 3.2 Å, the combination of *N*_B_ and Na^+^O6 shows a significant improvement with respect to the single CVs and underlines the importance of water in the binding region and the second to first shell transition around Na^+^. The highest predictive power is achieved by adding a CV that describes the surroundings of Cl^–^, but only with a minor increase.

**Table 1:** Best Predictive Power () of Linear Combinations of CVs for λ^r^ = 7.0 and λ^c^ = 3.2 Å (top) or λ^c^ = 3.7 Å (Bottom)^a^

	CV		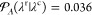
0.086	*N*_B_		
0.079	Na^+^O6		
0.103	Na^+^O6	–0.56 *N*_B_	
0.094	*N*_B_	+0.52 Na^+^O6H4	
0.108	Na^+^O6	–0.57 *N*_B_	–0.29 Cl^–^O1H4
0.107	Na^+^O6	+0.59 Cl^–^H7	–0.5 *N*_B_

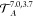	CV		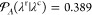
0.427	*N*_B_		
0.427	Na^+^O6		
0.442	Na^+^O6	–0.66 cos β_1_	
0.441	Na^+^O6	–0.72 cos α_1_	
0.446	cos α_1_	–0.74 Cl^–^O1Na^+^	+0.25 Na^+^O6
0.445	Na^+^O6	–0.81 cos β_1_	–0.39 Na^+^O5Cl^–^

a Each block presents the best two single CV, as well as the two best two- and three-CV combinations. The prefactors have been normalized by the prefactor with the highest absolute value.

Although it remains the best single CV at the transition state, *N*_B_ does not appear in the best linear combinations. It is replaced by the maximum cosine of the angles of all counter species of water in the first solvation shell around Cl^–^ (cos α_1_) and Na^+^(cos β_1_). The more maximal the cosine becomes, the closer it is located to the Na^+^–Cl^–^ axis. Therefore, in combination with Na^+^O6, the position and orientation of the water around the ion axis is now more discriminating than its quantity in the form of bridging water. Adding a third CV results in only a slight increase in predictive capacity compared to the linear combination based on two CVs. It should still be noted that in agreement with previous findings, the additional CVs provide more detailed information on the water arrangement around the Na^+^–Cl^–^ axis, particularly in the region between the two ions.

## 5 Conclusion

In this study, we investigated the mechanism and dynamics of NaCl dissociation using path sampling based on the RETIS algorithm. The analysis was conducted using the predictive power analysis (PPA) method, which evaluates the overlap integral of reactive and unreactive distributions along degrees of freedom orthogonal to the main order parameter. To enhance robustness, we refined the PPA methodology by applying a Savitzky-Golay (SG) filter to integrated distributions, enabling smooth high-resolution outputs while minimizing noise, which is characteristic of traditional binning-based methods.

Using this refined PPA method, we analyzed dissociation dynamics with the parameter set of Mullen et al. and additional orthogonal CVs derived from the index-invariant distance matrix (IIDM), with ionic distance λ as the main order parameter. Our findings revealed that the sixth closest oxygen to sodium (Na^+^O6) serves as an almost equally effective predictor of dissociation as the number of bridging waters (*N*_B_) from the Mullen et al. set. However, the IIDM set is simpler and is devoid of user biases. Nevertheless, our analysis identified intuitive parameters, showing that compression around sodium’s six closest water molecules strongly correlates with dissociation, while the solvent shell surrounding chloride is less significant.

Extending the analysis, we explored the predictive capacity of linear combinations of CVs by using the SG-based PPA method. Among these, the combination of Na^+^O6 and *N*_B_ provided the highest predictive power for two CVs, highlighting their complementary nature and the advantage of considering both simultaneously. Beyond the transition state, Na^+^O6 proved to be more effective than *N*_B_ for predicting progress toward the product state. Although adding a third CV yielded marginal improvements in predictive capacity, the additional complexity offers diminishing returns. Further analysis revealed that Na^+^O6 initially resides in the second solvation shell but migrates to the first shell during the reaction, forming an octahedral alignment with the other five closest oxygens around the sodium ion. This structural evolution offers valuable insights into the dissociation mechanism.

The refined PPA method, when combined with IIDM-derived CVs and human-selected nonlinear intuitive CVs, provides a systematic approach to identifying meaningful reaction coordinates for complex rare events. Compared to other CV discovery methods, such as likelihood maximization^10^ approaches or machine learning-based committor finding techniques,^18,42^ PPA has the advantage of interpretability and direct physical insights. While deep learning methods can uncover highly nonlinear CVs that correlate with a conceptual entity like the committor, they often require extensive training data and may lack transparency regarding the underlying physical mechanisms. In contrast, the PPA method is purely a posteriori method based on the analysis of a RETIS simulation that is used for rate evaluation, providing additional mechanistic insights in addition to quantitative numbers such as rate constants.

Moreover, committor-based analysis methods are most reliable in describing the separatrix or the committor 0.5 surface. However, understanding how the system progresses from a low committor value toward the separator is arguably even more important for obtaining mechanistic insights and identifying catalytic strategies. The PPA method also focuses on the very early stages of the reaction, where the committor is too small to be evaluated with standard shooting-type approaches. PPA provides meaningful and quantitative results via path reweighting and also by varying the λ^c^ and λ^r^ parameters, allowing different stages of the reaction to be targeted for analysis.

Yet, the above-mentioned approaches could, and probably should, be combined with PPA to maximize predictive power. As our results show, the IIDM-derived CVs, complemented by a set of human intuition-based CVs, leave the predictive capacity substantially below the theoretical optimum of one. Future enhancements could include incorporating momenta into the analysis or leveraging neural networks and symbolic regression^43^ to move beyond linear combinations, which have been pioneered in the committor-based analysis.^18,42^ However, increased complexity might compromise the interpretability of the results.

Beyond the current application, PPA has already been successfully applied to chemical reactions, such as water dissociation^12^ and even to learning efficiency based on students’ self-evaluation tests as an input.^44^ With the refined PPA method discussed in this paper, it could become a standard approach for studying complex transitions in systems, such as ionic liquids, ion channels, protein folding, self-assembly, and permeation. The PPA method applied to such processes is directly relevant for developing catalytic strategies and advancing drug discovery, as it provides detailed mechanistic insights into key processes, such as transport mechanisms, selectivity, and stability.

The PPA method could also be used to improve the sampling of driven rare events. Path sampling methods applicable to these nonequilibrium processes, such as forward flux sampling (FFS),^45^ adaptive multiple splitting (AMS),^46^ and weighted ensemble dynamics (WE),^47^ are typically restricted to forward-in-time propagation, making their efficiency more sensitive to the choice of RC. As a result, nonequilibrium rare events pose an even greater challenge than ordinary rare events. However, by combining PPA with a method like Contour FSS,^48^ the interfaces and RC could be optimized iteratively, enabling more efficient and reliable sampling of such events.

With the advent of faster RETIS algorithms^49−51^ and rapid advancements in machine learning techniques, including explainable AI,^52^ this methodology has the potential to evolve into a universal framework for obtaining intuitive insights from path sampling simulation data, which could ultimately be applied to guide complex processes in real-world experimental settings.

## Data Availability

The data that support the findings of this study are available from the corresponding author upon reasonable request.
